# Is the closest facility the one actually used? An assessment of travel time estimation based on mammography facilities

**DOI:** 10.1186/s12942-016-0039-7

**Published:** 2016-02-18

**Authors:** Jennifer Alford-Teaster, Jane M. Lange, Rebecca A. Hubbard, Christoph I. Lee, Jennifer S. Haas, Xun Shi, Heather A. Carlos, Louise Henderson, Deirdre Hill, Anna N. A. Tosteson, Tracy Onega

**Affiliations:** Department of Biomedical Science, Geisel School of Medicine at Dartmouth, Lebanon, NH USA; Department of Epidemiology, Geisel School of Medicine at Dartmouth, Lebanon, NH USA; Norris Cotton Cancer Center, Geisel School of Medicine at Dartmouth, Lebanon, NH USA; Group Health Research Institute, Seattle, WA USA; Department of Biostatistics and Epidemiology, Perelman School of Medicine, University of Pennsylvania, Philadelphia, PA 19104 USA; Department of Radiology, University of Washington School of Medicine, Seattle, WA USA; Department of Health Services, University of Washington School of Public Health, Seattle, WA USA; Division of General Internal Medicine and Primary Care, Brigham and Women’s Hospital, Boston, MA USA; The Geography Department, Dartmouth College, Hanover, NH USA; Department of Radiology, The University of North Carolina, Chapel Hill, NC USA; University of New Mexico, Albuquerque, NM USA; The Dartmouth Institute for Health Policy and Clinical Practice, Geisel School of Medicine at Dartmouth, Lebanon, NH USA

## Abstract

**Background:**

Characterizing geographic access depends on a broad range of methods available to researchers and the healthcare context to which the method is applied. Globally, travel time is one frequently used measure of geographic access with known limitations associated with data availability. Specifically, due to lack of available utilization data, many travel time studies assume that patients use the closest facility. To examine this assumption, an example using mammography screening data, which is considered a geographically abundant health care service in the United States, is explored. This work makes an important methodological contribution to measuring access—which is a critical component of health care planning and equity almost everywhere.

**Method:**

We analyzed one mammogram from each of 646,553 women participating in the US based Breast Cancer Surveillance Consortium for years 2005–2012. We geocoded each record to street level address data in order to calculate travel time to the closest and to the actually used mammography facility. Travel time between the closest and the actual facility used was explored by woman-level and facility characteristics.

**Results:**

Only 35 % of women in the study population used their closest facility, but nearly three-quarters of women not using their closest facility used a facility within 5 min of the closest facility. Individuals that by-passed the closest facility tended to live in an urban core, within higher income neighborhoods, or in areas where the average travel times to work was longer. Those living in small towns or isolated rural areas had longer closer and actual median drive times.

**Conclusion:**

Since the majority of US women accessed a facility within a few minutes of their closest facility this suggests that distance to the closest facility may serve as an adequate proxy for utilization studies of geographically abundant services like mammography in areas where the transportation networks are well established.

## Background

Appropriately assessing the influence that geographic access has on utilization of services is a critical component of health care planning and equity almost everywhere [1–4]. The ability to adequately measure the influence that geographic access has on health care service utilization depends on a broad range of methods available to researchers and the healthcare context for which the method is applied [5–11]. Globally popular methods for characterizing geographic access include the two step floating catchment area (2SFCA), areal interpolation methods such as kernel density estimation (KDE), and travel time studies [6, 7]. Notably, comparative analyses of these methods demonstrates that there is no conclusive, standard approach to characterizing geographic access in the context of healthcare; and because of this lack of standard it is difficult to determine the true magnitude of potential effect that geographic access has on utilization or healthcare services regardless of the geographic locale of study [6, 7, 12].

Since travel time is identified as a frequently used measure of geographic access to health care services, we will examine one of the fundamental assumptions associated with this particular geographic access method citing mammography in the United States as an example [13–30]. Typically, travel time is calculated as a driving time either based on geocoded residential addresses or, more commonly, using the distance from a representative residential location (e.g. polygon-based calculated centroids such as census block/block group/tract and/or ZIP code centroid in the United States) to the facility. Many travel time studies assume that individuals access health care services at the facility closest to the location where they reside [9, 12–15, 20, 21, 23, 24, 31, 32]. Thus, reported travel times have represented *minimum travel times* that may differ from *actual travel times* based on health care service utilization data.

Further, differences in potential versus actual travel time may vary by population subgroup characteristics in the United States. For example, rural patients may be more likely to use the facility that is closest because of fewer available service options or may be influenced by non-geographic factors such as seasonal weather burdens [22]. To our knowledge, prior studies have not tested the assumption that patients use the closest facility or whether this assumption differs for some population subgroups. This work addresses a fundamental methodological question in spatial epidemiology and health services research: concordance of proximity versus utilization-based measures of health care access, and whether population characteristics modify concordance.

We focus on mammography in the United States which is a relatively geographically abundant health care service. Prior US based work has measured geographic access to breast imaging facilities in terms of travel time to the closest facility [7, 14, 15, 19, 21, 24, 33]. However, these studies did not examine utilization data and thus were not able to fully characterize individuals’ travel patterns. Research suggests that, despite the abundance of mammography facilities, there are sub-populations for whom utilization is lower than recommended [23, 24, 27, 34, 35]. Increased travel burden may contribute to lower utilization rates in these sub-populations, but this hypothesis is difficult to assess when measures of geographic access are typically based solely on proximity to closest services [14, 18, 33, 36] rather than the services actually used.

We compare travel times between the closest and actual mammography facility attended for women within five registries of the Breast Cancer Surveillance Consortium (BCSC) [37] to establish the magnitude of differences between the two. By linking mammography registry data with patient and population characteristics from US Census data, we are able to quantify and compare closest versus actual geographic access for population subgroups, including stratifying patients by age, race, rural residence, and income. We also examine individual facility characteristics in order to determine if there may be health system factors associated with women more willing to travel further in order to obtain services.

## Methods

### Study population

Our study population includes all women aged 30–90 years who received a mammography exam at a facility participating in the BCSC between 2004 and 2010. In order to avoid over-estimating travel times, only one mammogram was included per woman. If there were multiple exams per woman, we randomly selected one. The BCSC is a National Cancer Institute (NCI)-funded network of mammography registries across the United States. We used registry data from New Hampshire, North Carolina, San Francisco, Vermont, and New Mexico. Each registry, as well as the Statistical Coordinating Center (SCC) that processed the pooled data, has received institutional review board (IRB) approval for either active or passive consenting processes or a waiver of consent to enroll participants, link data, and perform analytic studies. All procedures are Health Insurance Portability and Accountability Act (HIPAA) compliant and all registries and the SCC have received a Federal Certificate of Confidentiality and other protection for the identities of women, physicians, and facilities involved in this research.

### Patient and facility characteristics

At the time of examination, women receiving mammography at a BCSC facility self-report basic demographic information including age, ethnicity/race and highest level of education. For women in the BCSC with multiple mammography exams, we randomly selected one exam per woman. We linked individual self-report demographic data to community-level characteristics that were obtained through address-based linkage to 2010 US Census data from the ESRI Business Analyst application [38]. Community-level demographic information included rural–urban status, diversity index score [a measure of the population diversity of a given geographic area that ranges from 0 (low diversity) to 1 (high diversity)] [39], median household income level, median travel time to work, and mean dollars spent on public transportation per year. The lowest level of geography available, either census block group, tract or ZIP code level, for each variable is indicated in Table 1.

**Table 1:** Sample characteristics of BCSC mammograms by woman, facility, and census-level characteristics

	Geographic level of variable	N	%	N missing
Total population		646,553		
Woman-level characteristics				
Age	Woman level			0
30–40		35,483	5.5	
40–49		194,760	30.1	
50–59		181,832	28.1	
60–69		126,106	19.5	
70–79		75,192	11.6	
80+		33,180	5.1	
Race/ethnicity	Woman level			119,624
White, non-Hispanic		372,016	70.6	
Black, non-Hispanic		38,949	7.4	
Hispanic		27,315	5.2	
Asian		75,278	14.3	
Native American		3040	0.6	
Other		10,331	2	
Education	Woman level			242,322
Less than high school		37,773	9.3	
High school		83,888	20.8	
Some college		100,696	24.9	
College or post-college graduate		181,874	45	
Census-level^b^ characteristics				
Rurality	Census^b^: 2006 zip code			238
Urban core		452,224	70	
Suburban areas		41,888	6.5	
Large town areas		93,165	14.4	
Small town and isolated rural		59,038	9.1	
Median household income	Census^b^: Block group			503
<45K		144,480	22.4	
45–59,999K		139,912	21.7	
60–84,999K		198,303	30.7	
85K+		163,355	25.3	
Diversity index	Census^b^: Tract			503
0–25		148,217	22.9	
26–50		158,971	24.6	
51–75		243,632	37.7	
76–100		95,230	14.7	
Average spent on public transportation	Census^b^: Block group		503	
<100$		364,469	56.4	
>100$		281,581	43.6	
Median travel time to work	Census^b^: Block group			3
<15 min		51,038	7.9	
15–30 min		473,988	73.3	
>30 min		121,524	18.8	
Actual facility^a^ characteristics				
Academic status	Facility level^a^			172,334
Academic		65,966	13.9	
Non-academic		408,253	86.1	
Practice type	Facility level^a^			172,334
Multispecialty		126,331	26.6	
General radiology		330,449	69.7	
Breast imaging only		13,370	2.8	
Non-radiology		4069	0.9	

^a^Facility where the exam took place

^b^Census-level variable for woman’s place of residence

In 2012, registries provided data on characteristics of each BCSC participating mammography facility. This analysis provided results from 105 of the BCSC affiliated locations. These data included academic affiliation (academic, non-academic) and practice type (multi-specialty breast center, radiology, breast imaging only, and non-radiology). A “multi-specialty breast center” refers to a practice with additional specialties beyond radiology (e.g., cancer center with breast imagers, breast surgeons, and breast oncologists); “general radiology” practice images other body parts in addition to the breasts; “breast imaging only” is a radiology practice only imaging the breasts; and “non-radiology” is a facility without radiologists that offers screening mammography services on-site (e.g., an obstetrician-gynecologist clinic).

### Closest and actual travel times

To calculate travel time, we obtained residential and individual mammography facility addresses and geocoded them to the street level. Approximately 10 % of patient addresses could not be geocoded at the street address level and were excluded from the analysis. Since some facilities in a woman’s area may not participate in the BCSC, each registry provided a catchment area to search for additional locations that provide mammography. To delineate a catchment area, each registry provided a list of zip codes that are considered part of the facility catchment area. Those zip codes were then used to look up facilities listed in the Food and Drug Administration (FDA) mammography database [40] that do not participate in the BCSC. The BCSC versus non-BCSC counts for each registry are listed here: in NC, we had 236 BCSC facilities and 5 non-BCSC facilities, NH we have 47 BCSC and 11 non-BCSC facilities, San Francisco we have 25 BCSC facilities and 98 non-BCSC facilities, NM has 27 BCSC facilities and 30 non-BCSC facilities, and VT has 16 BCSC facilities and 1 non-BCSC facility. This is a total of 321 BCSC facilities and 145 non-BCSC facilities for our study population. For each registry, we used ArcGIS v. 10.1 (Environmental Systems Research Institute, Redlands, CA), and the Streetmap N.A. network datasest, to calculate the shortest travel time between each woman’s address and facility location pair, with 180 min as the upper limit. We exclude patients outside of the 180 min, or if their residential address was outside of the designated facility catchment area, to reduce computational challenges. We chose a 180 min cut off since we assume it is unlikely a woman would drive more than 3 h for a mammography screening. Outside of catchment area excludes N = 9810 women; outside the 180 min service area excludes an additional 6485 women. Differential drive time was calculated as the difference between drive time to the closest facility and the facility actually used. A differential drive time of 0 indicates that the exam took place at the closest facility; differences greater than 0 indicate that the woman did not use the closest facility for the exam.

### Statistical analysis

We summarized the actual, closest and differential drive times by sub-groups defined by patient and facility characteristics using univariate summary statistics [median and interquartile range (IQR)]. Additionally, we calculated the proportion of women using the closest facility including cumulative distribution functions for closest, actual, and differential drive time, accounting for censoring of actual drive times at 180 min. The probability of differential drive time being less than 5, 10, 30 and 60 min for different covariate levels were derived from the cumulative distribution functions. These univariate summary measures enable us to describe whether the difference between travel times to the closest and actual facility differs across population sub-groups. All statistical analyses were conducted in R 3.1.2 [41].

## Results

### Patient- and facility-level characteristics

The 646,553 eligible mammography exams in our study sample are described in Table 1. Overall, the largest proportion of women in the sample population were within the screening ages of 40–49 (30.1 %), 50–59 (28.1 %) and 60–69 (19.5 %). 70.6 % of the women were non-Hispanic white, and the largest proportion of the sample population reported at least college or post-graduate degrees (45 %). Most exams took place at non-academic facilities (86.1 %) and general radiology practices (69.7 %).

### Area-level characteristics

Census characteristics reveal that the sample population lived in predominantly ‘Urban Core’ (70 %) areas. The median household income was relatively high in this sample population. For example, 30.7 % of the Census block groups reported a median household income of ‘60–84K’ and 25.3 % reported an income of ‘85K or higher’. More than half of the sample lived in areas in which residents spent less than $100 a year on public transportation (56.4 %). Moreover, nearly three quarters of study participants lived in Census block groups where the median travel time to work was ‘15–30 min’ and 18.8 % % lived in areas having a median travel time to work ‘greater than 30 min’.

### Travel time summary

**Fig. 1:**
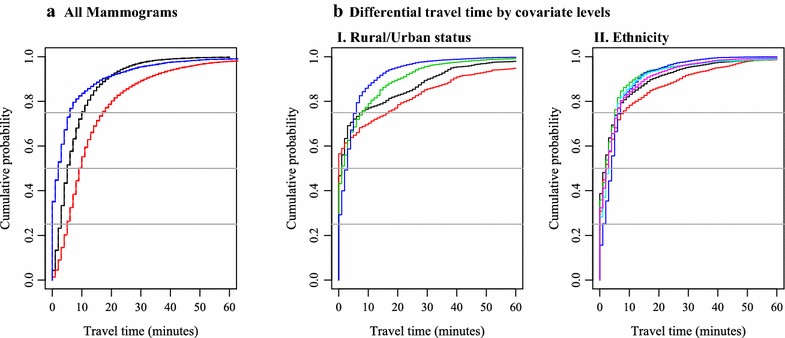
**a** Cumulative distribution functions of actual, closest, and differential drive time. **b** Cumulative distribution functions of differential drive times by covariates. **I** Rural/urban status. **II** Ethnicity. **a** Closest. Actual. Differential. **bI** Large rural town. Small town/isolated rural. Sub-urban. Urban core. **bII** White, non-Hispanic. Black, non-Hispanic. Hispanic. Asian. Native American. Other

The travel times to the closest and actual facilities visited are summarized in Table 2. Figure 1 shows the cumulative distribution of closest, actual, and differential travel time overall and stratified by population characteristics. The median travel time to the closest facility was 5 min (IQR [3,10]), and to the actual facility was 9 min (IQR [5,17]). Rural/urban status revealed notable differences in travel time to closest and actual facilities. Those living in the urban core had a median travel time to the closest facility of 4 min (IQR [2,7]), while those living in small towns or isolated rural areas had a median travel time of 14 min to the closest facility (IQR [6,25]) and an actual travel time of 23 min (IQR [9,41]).

**Table 2:** Summary of closest, actual, and differential travel times in sample of BCSC mammograms by woman, facility, and census-level characteristics

	Geographic level of variable	Travel time-median and IQR		Cumulative distribution of differential travel time (proportion)			
		Closest facility	Actual facility	(actual = closest) 0 m	<5 m	<10 m	<30 m
All		5 (3,10)	9 (5,17)	0.35	0.73	0.84	0.96
Woman-level characteristics							
Age	Woman level						
30–40		6 (3,11)	11 (6,19)	0.29	0.68	0.79	0.94
40–49		5 (3,10)	10 (6,18)	0.33	0.7	0.82	0.96
50–59		6 (3,11)	10 (6,18)	0.34	0.72	0.83	0.95
60–69		5 (3,11)	9 (5,17)	0.37	0.74	0.84	0.95
70–79		5 (2,9)	8 (4,15)	0.40	0.79	0.88	0.96
80+		4 (2,8)	7 (4,12)	0.44	0.83	0.91	0.98
Race/ethnicity	Woman level						
White, non-Hispanic		6 (3,12)	10 (6,19)	0.39	0.74	0.83	0.95
Black, non-Hispanic		5 (3,10)	10 (6,21)	0.31	0.68	0.78	0.92
Hispanic		6 (3,10)	9 (6,14)	0.36	0.76	0.89	0.97
Asian		3 (2,5)	8 (4,11)	0.16	0.66	0.86	0.98
Native American		5 (3,10)	10 (6,16)	0.28	0.73	0.87	0.96
Other		4 (2,8)	8 (4,16)	0.32	0.72	0.84	0.97
Education	Woman level						
Less than high school		4 (2,7)	8 (4,12)	0.37	0.77	0.90	0.97
High school		6 (3,13)	10 (5,19)	0.43	0.75	0.85	0.95
Some college		5 (3,10)	9 (5,18)	0.37	0.72	0.82	0.95
College or post-college graduate		4 (2,9)	9 (5,16)	0.31	0.71	0.83	0.96
Census-level^b^ characteristics							
Rurality	Census^b^: 2006 zip code						
Urban core		4 (2,7)	8 (5,13)	0.29	0.75	0.87	0.98
Suburban areas		18 (12,24)	23 (15,32)	0.43	0.69	0.79	0.96
Large town areas		9 (4,16)	14 (6,27)	0.47	0.72	0.77	0.9
Small town and isolated rural		14 (6,25)	23 (9,41)	0.57	0.65	0.7	0.86
Median household income	Census^b^: Block group						
<45K		6 (3,14)	10 (5,25)	0.43	0.73	0.8	0.92
45–59,999K		8 (3,14)	12 (6,23)	0.38	0.72	0.81	0.94
60–84,999K		5 (3,9)	9 (6,15)	0.34	0.74	0.88	0.98
85K+		4 (2,7)	8 (5,14)	0.28	0.72	0.85	0.98
Diversity index	Census^b^: Tract						
0–25		11 (5,18)	14 (7,24)	0.53	0.76	0.83	0.95
26–50		6 (2,10)	9 (5,17)	0.35	0.74	0.82	0.95
51–75		4 (2,7)	8 (5,14)	0.27	0.72	0.85	0.96
76–100		4 (2,8)	9 (5,14)	0.28	0.69	0.83	0.97
Average spent on public transportation	Census^b^: Block group						
<100$		8 (4,14)	12 (7,22)	0.38	0.72	0.82	0.93
>100$		3 (2,6)	7 (4,12)	0.31	0.75	0.86	0.98
Median travel time to work	Census^b^: Block group						
<15 min		4 (2,6)	6 (3,15)	0.54	0.78	0.8	0.88
15–30 min		6 (3,11)	10 (6,17)	0.36	0.74	0.84	0.96
>30 min		5 (3,9)	9 (6,19)	0.22	0.69	0.85	0.98
Actual facility^a^ characteristics							
Academic status	Facility level^a^						
Academic		4 (2,9)	9 (5,17)	0.31	0.69	0.83	0.95
Non-academic		4 (2,8)	9 (5,14)	0.33	0.75	0.87	0.98
Practice type	Facility level^a^						
Multispecialty		3 (2,6)	8 (4,14)	0.22	0.68	0.83	0.98
General radiology		5 (2,9)	9 (5,15)	0.38	0.77	0.88	0.97
Breast imaging only		7 (4,11)	10 (7,16)	0.22	0.72	0.87	0.96
Non-radiology		8 (4,14)	12 (7,21)	0.42	0.73	0.85	0.98

^a^Facility where the exam took place

^b^Census-level variable for woman’s place of residence

### Summary of differential travel times

Overall, 65 % of exams did not occur at a woman’s closest facility (Table 2). Women who were older (44 % of women 80 and older), lived in non-urban areas (57 % of small town and isolated rural), and/or areas with low diversity (53 % of 0–25 diversity index) or lower median incomes (43 % of 45K or less) were more likely to use the closest facility. These exams were more likely to take place at a ‘general radiology’ (38 %) or ‘non-radiology’ (42 %) location.

In contrast, exams that were less likely to take place at the closest facility included those obtained at a “breast imaging only” (22 %) or “multispeciality” (22 %) facility and those for women who lived in census blocks in urban core areas (29 %), with higher population diversity indices (27 % of areas with 51–75 % diversity score), greater median yearly income (28 % for 85K or higher), and/or longer median travel times to work (22 % for a commute of 30 min or longer).

While approximately one-third of exams took place at the closest facility, most occurred at facilities that were within a few minutes’ drive of the closest facility. Figure 1 shows the cumulative distribution function for differential travel time. Overall, 73 % of exams took place at facilities at or within 5 min’ travel time of the closest facility. This percentage displayed relatively little variability across subgroups (all were in a range of ±10 % points).

Specific sub-groups for which comparatively more exams took place within 5 min of the closest facility included women >80 years old (83 %) and those living in a block group with a median travel time to work <15 min (78 %). Sub-groups for which a smaller proportion of exams had differential drive times ≤5 min included women who were Black or Asian (68 and 66 %, respectively), women living in an area with high population diversity (69 % of 76–100 % diversity score), women <40 years old (68 % of women ages 30–40), women living in a suburban area (69 %), and women living in areas where the median travel time to work was >30 min (69 %).

Figure 1b provides a more detailed view of the differences by rural/urban status and ethnicity. We selected these specific variables because they highlight most noticeable differences in differential drive times. Particularly, exams obtained in urban cores were less likely to take place at the closest facility but overall had relatively small differential drive times, compared with suburban and rural regions. The distribution of differential drive times is quite similar by ethnicity, although there is some indication that the distribution is slightly different among African American women.

## Discussion

Our study provides an improved understanding of how accuracy in measuring geographic access is likely to matter in the broader context of healthcare utilization, regardless of study locale. Specifically, our study critically examines a common assumption associated with estimating geographic access—whether or not using the closest facility to evaluate geographic access is an adequate proxy for utilization data. Our analysis aimed to elucidate the differences that may exist between closest and actual facility attended both overall and for specific populations, and contexts. To our knowledge, this type of comparative analysis has not been applied to this common assumption before.

Our analysis was facilitated by having both the street level addresses of patients and mammography facilities as well as utilization data from the nationally representative BCSC sample of breast imaging facilities. Our study reveals that only 35 % of women undergoing mammography used the closest facility. Nevertheless, nearly 75 % of women undergoing mammography used a facility within 5 min of the closest facility. Population-level urban/rural status, income, and travel time to work appear to be more closely associated with the use of the closest facility. In general, these findings point to the fact that those living in more diverse urban areas and those traveling farther for work are less likely to use their closest facilities.

Our study suggests that appropriateness of using the common assumption that travel time to the closest facility to assess geographic access to healthcare service may depend on the abundance of the service. In an area where the service is geographically abundant, people may be less likely to use the closest facility; whereas in areas where the services are less uniformly available, including rural areas or areas that are far from places of employment, the assumption of closest facility utilization is more likely to be valid.

Nonetheless, analyses for which closest travel time is used as an approximation to determine the influence geographic access has on utilization may not be completely in error given that in the United States nearly three-fourths of women not utilizing the closest facility in this sample traveled to facilities within 5 min of the closest facility. This means that for geographically abundant services like mammography, even if they are not choosing their closest facility, the facility they are choosing is unlikely to be dramatically distant from their closest facility. However, in analyses where this approximation is used as a predictor, this will introduce measurement error which can lead to bias when estimating relationships between access to care and health outcomes [42].

It is also important to note that the interquartile ranges of the actual travel times were quite large in many sub-groups of our study population, even though the median values were relatively small (e.g., women younger than 40 had an actual median travel time of 10, but the IQR 6–18). For some, their round trip travel time may be substantially longer than the median for this study sample. Specifically, this analysis only reflects a one-way travel time, and to get a more accurate picture of the total travel burden we would need to obtain a round trip travel time. For this example, median round trip travel to a mammographic service was 20 min with an interquartile range of 12–36 min. This is important to consider when evaluating the differential travel times since the discrepancies between travel time to the closest and actual facilities would be larger in analyses that examined round trip travel times.

The results of this paper augment existing US based studies in which the influence of geographic access to healthcare, as measured by travel time, is not precisely understood [13, 15, 33]. Often this is due to limitations in available data that compel researchers to use the closest facility assumption in order to gain insight into health care utilization patterns [7, 14, 19, 21, 36]. Our study reveals that under certain conditions, the use of the closest facility to estimate geographic access to mammographic facilities may be appropriate. In the pooled data, we found that the closest facility assumption is most likely to hold for women living in rural communities and in populations that are older, have lower socio-economic status and are more racially and ethnically homogeneous.

A more precise model of utilization facilitates insight into the role that travel time plays in choice of health care facility, particularly for breast cancer screening in the context of the United States. In our study, it appears that with a relatively geographically abundant health service such as mammography, geographic access is generally longer for certain rural sub-populations. For example, for almost all of those in an urban core, a facility can be reached in <30 min; whereas, in a small town and isolated rural areas, that number climbs to >60 min. It appears that urban residents, more so than their rural counterparts, may be able to exercise “choice” in facilities due to the relative abundance of mammographic services within a small geographic area.

The translation of these results to international settings outside of the United States are broadly applicable to geographic locales that maintain similar geographic features to our example in relation to (1) the availability of abundant health care services (such as our case with mammography), (2) with well-established transportation networks.

It is especially important to note that the apparent rural access burden in the United States may be tempered by the fact that individuals who work further away from their residences may actually have geographic access patterns that align with their regular daily routines. For instance, the potential burden would be lessened if a woman can access the service at a location near her place of employment. This general observation could also apply to women outside the United States that travel reasonably far distances to work and want to obtain care close to her place of employment to minimize the daily travel burden.

Limitations of our analysis include the assumption that all registry patients have personal access to a vehicle. This assumption persists in most travel time analyses [7, 15, 19–23, 31, 33, 35, 36] that have indicated that urban travel times are relatively small, but assume that individuals are driving a single passenger vehicle when they are likely walking or taking public transportation to their destination. Future studies should accommodate urban multi-modal transportation networks. A study by Peipins et al. [23], which included a multi-modal network for Atlanta, further illustrates the need for national multi-modal networks to include both public and private transportation. The need for a multi-modal transportation network is of particular relevance to the international community for which multi-modal transportation networks are relatively more broadly accepted as a means of travel than the United States which is commonly associated with single passenger travel. Additionally, while the BCSC sample data are considered representative of the US population, this analysis does not include Hawaii and Alaska which have larger proportions of rural populations and different modes of transportation including water or air transportation, both resulting in different patterns of access and utilization compared to other sub-populations [27]. Moreover, the highest proportion of women represented in this study coincides with the time that most women begin screening (age 40–49), and within this study population, the data reflect a large proportion of women who are highly educated with a high income (two variables that tend to coincide with one another). Additionally, for median household income, rurality, population diversity index, dollars spent on public transportation and median travel time to work, we used population-level data from the US Census as individual-level data were not available, which may lead to ecological fallacy. Finally, this study is descriptive in nature and focuses on univariate relationships. Indeed, many of the demographic factors investigated are likely correlated with geographic location/rurality.

Two strengths of this paper are the availability of utilization data, and improved accuracy of using geocoded street level addresses for both women’s residences and breast imaging facility locations to calculate travel times. To our knowledge, no other health services research study has been able to achieve this level of detail in travel time analysis to examine utilization patterns either in the United States or globally. Additional strengths of the study are the large sample size, as well as the rich information about characteristics of women, facilities, and the environment.

As a final point, this study does not examine clinical factors that might alter the relationship between proximity and utilization. For instance, for women with a cancer diagnosis, proximity may be an essential consideration due to frequency of visits or intensity of subsequent treatment regimens. This could change the relationship between patient and facility characteristics and differential drive time. This remains an opportunity to expand on the current understanding of the underlying factors that affect mammography utilization.

## Conclusion

Unlike previous travel time studies, this analysis is able to examine geographic access patterns using both patient level and breast imaging facility level street addresses and then compare the common closest facility assumption against actual utilization patterns. We found that a large proportion of our US based sample that didn’t use the closest facility, roughly three-quarters, accessed care at a facility within 5 min of their closest facility, suggesting that use of the closest facility may not introduce substantial error for studies of geographically abundant services in areas where the transportation network is well established. Therefore, the use of the closest facility as a proxy to summarize geographic access appears to be appropriate for certain population sub-groups. Where populations are by-passing the closest facility, it appears that individuals tend to live in an urban core, or may have greater income, or include areas where the travel time to work might be longer. Additional research is needed to investigate whether similar relationships hold in the context of other health care services outside of the United States, particularly those that are not as geographically dense or where populations access healthcare facilities by other transportation means such as walking, riding a bike or by public transit.
